# Supplementary material for: Evaluation of waterlogging tolerance and responses of protective enzymes to waterlogging stress in pumpkin
Source: PeerJ. 2023 Apr 21;11:e15177. doi: 10.7717/peerj.15177 (PMC10124548; doi:10.7717/peerj.15177)
Supplement: Supplemental Information 1 [file peerj-11-15177-s001.docx]

| treat day | viatery | blank1 | blank2 | △CK | measure | CK | △A | percentage inhibition | SOD（U/g） |
| --- | --- | --- | --- | --- | --- | --- | --- | --- | --- |
|  |  | CK1 | CK2 | △CK | A1 | A2 | △A | B |  |
|  |  |  |  | △CK=CK1-CK2 |  |  | △A=A1-A2 | B=(△CK-△A)/△CK | SOD=11.11×B/(1-B)/0.1 |
| 0d | 8-1 | 0.584 | 0.225 | 0.359 | 0.216 | 0.041 | 0.175 | 0.512534819 | 116.81371 |
|  | 8-2 | 0.584 | 0.225 | 0.359 | 0.203 | 0.041 | 0.162 | 0.548746518 | 135.10309 |
|  | 8-3 | 0.584 | 0.225 | 0.359 | 0.233 | 0.041 | 0.192 | 0.465181058 | 96.63385 |
|  |  |  |  |  |  |  |  |  | 116.18355 |
|  | 10-1 | 0.584 | 0.225 | 0.359 | 0.246 | 0.073 | 0.173 | 0.51810585 | 119.44855 |
|  | 10-2 | 0.584 | 0.225 | 0.359 | 0.247 | 0.073 | 0.174 | 0.515320334 | 118.12356 |
|  | 10-3 | 0.584 | 0.225 | 0.359 | 0.243 | 0.073 | 0.17 | 0.526462396 | 123.51706 |
|  |  |  |  |  |  |  |  |  | 120.36306 |
| 1d | 8-1 | 0.584 | 0.225 | 0.359 | 0.256 | 0.048 | 0.208 | 0.420612813 | 80.65433 |
|  | 8-2 | 0.584 | 0.225 | 0.359 | 0.258 | 0.048 | 0.21 | 0.415041783 | 78.82810 |
|  | 8-3 | 0.584 | 0.225 | 0.359 | 0.257 | 0.048 | 0.209 | 0.417827298 | 79.73684 |
|  |  |  |  |  |  |  |  |  | 79.73975 |
|  | 10-1 | 0.584 | 0.225 | 0.359 | 0.242 | 0.062 | 0.18 | 0.498607242 | 110.48278 |
|  | 10-2 | 0.584 | 0.225 | 0.359 | 0.218 | 0.062 | 0.156 | 0.56545961 | 144.57244 |
|  | 10-3 | 0.584 | 0.225 | 0.359 | 0.261 | 0.062 | 0.199 | 0.445682451 | 89.32663 |
|  |  |  |  |  |  |  |  |  | 114.79395 |
| 3d | 8-1 | 0.584 | 0.225 | 0.359 | 0.224 | 0.064 | 0.16 | 0.554317549 | 138.18063 |
|  | 8-2 | 0.584 | 0.225 | 0.359 | 0.235 | 0.064 | 0.171 | 0.52367688 | 122.14503 |
|  | 8-3 | 0.584 | 0.225 | 0.359 | 0.218 | 0.064 | 0.154 | 0.571030641 | 147.89286 |
|  |  |  |  |  |  |  |  |  | 136.07284 |
|  | 10-1 | 0.584 | 0.225 | 0.359 | 0.15 | 0.025 | 0.125 | 0.651810585 | 207.97920 |
|  | 10-2 | 0.584 | 0.225 | 0.359 | 0.14 | 0.025 | 0.115 | 0.679665738 | 235.72522 |
|  | 10-3 | 0.584 | 0.225 | 0.359 | 0.146 | 0.025 | 0.12 | 0.665738162 | 221.27417 |
|  |  |  |  |  |  |  |  |  | 221.65953 |
| 5d | 8-1 | 0.584 | 0.225 | 0.359 | 0.363 | 0.083 | 0.28 | 0.22005571 | 31.34607 |
|  | 8-2 | 0.584 | 0.225 | 0.359 | 0.285 | 0.083 | 0.202 | 0.437325905 | 86.35000 |
|  | 8-3 | 0.584 | 0.225 | 0.359 | 0.32 | 0.083 | 0.237 | 0.339832869 | 57.19072 |
|  |  |  |  |  |  |  |  |  | 58.29560 |
|  | 10-1 | 0.584 | 0.225 | 0.359 | 0.321 | 0.173 | 0.148 | 0.587743733 | 158.39257 |
|  | 10-2 | 0.584 | 0.225 | 0.359 | 0.338 | 0.177 | 0.161 | 0.551532033 | 136.63230 |
|  | 10-3 | 0.584 | 0.225 | 0.359 | 0.337 | 0.174 | 0.163 | 0.545961003 | 133.59264 |
|  |  |  |  |  |  |  |  |  | 142.87250 |
| 7d | 8-1 | 0.584 | 0.225 | 0.359 | 0.29 | 0.129 | 0.161 | 0.551532033 | 136.63230 |
|  | 8-2 | 0.584 | 0.225 | 0.359 | 0.307 | 0.129 | 0.178 | 0.504178273 | 112.97247 |
|  | 8-3 | 0.584 | 0.225 | 0.359 | 0.296 | 0.129 | 0.167 | 0.534818942 | 127.73174 |
|  |  |  |  |  |  |  |  |  | 125.77884 |
|  | 10-1 | 0.584 | 0.225 | 0.359 | 0.2 | 0.054 | 0.146 | 0.593314763 | 162.08425 |
|  | 10-2 | 0.584 | 0.225 | 0.359 | 0.198 | 0.054 | 0.144 | 0.598885794 | 165.87847 |
|  | 10-3 | 0.584 | 0.225 | 0.359 | 0.214 | 0.054 | 0.16 | 0.554317549 | 138.18063 |
|  |  |  |  |  |  |  |  |  | 155.38111 |

|  |  | 8-1 | 8-2 | 8-3 |  |  |
| --- | --- | --- | --- | --- | --- | --- |
|  | 0d | 116.8137143 | 135.1030864 | 96.63385417 | 116.1835516 |  |
|  | 1d | 80.65432692 | 78.82809524 | 79.73684211 | 79.73975476 |  |
|  | 3d | 138.180625 | 122.1450292 | 147.8928571 | 136.0728371 |  |
|  | 5d | 31.34607143 | 86.35 | 57.1907173 | 58.29559624 |  |
|  | 7d | 136.6322981 | 112.9724719 | 127.7317365 | 125.7788355 |  |
|  |  |  |  |  |  |  |
|  |  | 10-1 | 10--2 | 10-3 |  |  |
|  | 0d | 119.4485549 | 118.1235632 | 123.5170588 | 120.363059 |  |
|  | 1d | 110.4827778 | 144.5724359 | 89.32663317 | 114.7939489 |  |
|  | 3d | 207.9792 | 235.7252174 | 221.2741667 | 221.659528 |  |
|  | 5d | 158.3925676 | 136.6322981 | 133.592638 | 142.8725012 |  |
|  | 7d | 162.0842466 | 165.8784722 | 138.180625 | 155.3811146 |  |
|  |  |  |  |  |  |  |
|  |  |  |  |  |  |  |
| The letter marks indicate the result |  |  |  |  |  |  |
| treat | average | 5%significant levels | treat | average | SE |  |
| 10-3 | 221.6595 | a | 8-0 | 116.1836 | 15.7113 |  |
| 10-7 | 155.3811 | b | 8-1 | 79.7398 | 0.7456 |  |
| 10-5 | 142.8725 | bc | 8-3 | 136.0728 | 10.6166 |  |
| 8-3 | 136.0728 | bcd | 8-5 | 58.2956 | 22.4688 |  |
| 8-7 | 125.7788 | cd | 8-7 | 125.7788 | 9.7573 |  |
| 10-0 | 120.3631 | d | 10-0 | 120.3631 | 2.2949 |  |
| 8-0 | 116.1836 | d | 10-1 | 114.7939 | 22.7591 |  |
| 10-1 | 114.7939 | d | 10-3 | 221.6595 | 11.3305 |  |
| 8-1 | 79.7398 | e | 10-5 | 142.8725 | 11.0443 |  |
| 8-5 | 58.2956 | f | 10-7 | 155.3811 | 12.2608 |  |
|  |  |  |  |  |  |  |
|  | 0 | 1 | 3 | 5 | 7 |  |
| Baimi 8 | 116.1835516 | 79.73975476 | 136.0728371 | 58.84803571 | 125.7788355 |  |
| Baimi 10 | 120.363059 | 114.7939489 | 221.659528 | 142.8725012 | 155.3811146 |  |
|  |  |  |  |  |  |  |
