# Supplementary material for: Evaluation of waterlogging tolerance and responses of protective enzymes to waterlogging stress in pumpkin
Source: PeerJ. 2023 Apr 21;11:e15177. doi: 10.7717/peerj.15177 (PMC10124548; doi:10.7717/peerj.15177)
Supplement: Supplemental Information 2 [file peerj-11-15177-s002.docx]

| treat day | variety | A30s | A90s | △A | POD(U/g) |
| --- | --- | --- | --- | --- | --- |
|  |  |  |  | △A=A90s-A30s | POD=4900*△A/0.1 |
| 0d | 8-1 | 0.073 | 0.174 | 0.101 | 4949 |
|  | 8-2 | 0.086 | 0.187 | 0.101 | 4949 |
|  | 8-3 | 0.128 | 0.232 | 0.104 | 5096 |
|  |  |  |  |  | 4998 |
|  | 10-1 | 0.096 | 0.182 | 0.086 | 4214 |
|  | 10-2 | 0.105 | 0.189 | 0.084 | 4116 |
|  | 10-3 | 0.164 | 0.249 | 0.085 | 4165 |
|  |  |  |  |  | 4165 |
| 1d | 8-1 | 0.209 | 0.332 | 0.123 | 6027 |
|  | 8-2 | 0.138 | 0.272 | 0.134 | 6566 |
|  | 8-3 | 0.075 | 0.202 | 0.127 | 6223 |
|  |  |  |  |  | 6272 |
|  | 10-1 | 0.138 | 0.259 | 0.121 | 5929 |
|  | 10-2 | 0.132 | 0.241 | 0.109 | 5341 |
|  | 10-3 | 0.132 | 0.241 | 0.109 | 5341 |
|  |  |  |  |  | 5537 |
| 3d | 8-1 | 0.792 | 1 | 0.208 | 10192 |
|  | 8-2 | 0.908 | 1.151 | 0.243 | 11907 |
|  | 8-3 | 0.585 | 0.822 | 0.237 | 11613 |
|  |  |  |  |  | 11237.33333 |
|  | 10-1 | 0.79 | 0.991 | 0.201 | 9849 |
|  | 10-2 | 0.675 | 0.878 | 0.203 | 9947 |
|  | 10-3 | 1.144 | 1.332 | 0.188 | 9212 |
|  |  |  |  |  | 9669.333333 |
| 5d | 8-1 | 1.791 | 2.016 | 0.225 | 11025 |
|  | 8-2 | 1.798 | 2.026 | 0.228 | 11172 |
|  | 8-3 | 1.019 | 1.244 | 0.225 | 11025 |
|  |  |  |  |  | 11074 |
|  | 10-1 | 1.268 | 1.464 | 0.196 | 9604 |
|  | 10-2 | 1.606 | 1.807 | 0.201 | 9849 |
|  | 10-3 | 0.726 | 0.932 | 0.206 | 10094 |
|  |  |  |  |  | 9849 |
| 7d | 8-1 | 1.811 | 1.911 | 0.1 | 4900 |
|  | 8-2 | 1.814 | 1.91402 | 0.10002 | 4900.98 |
|  | 8-3 | 1.847 | 1.948 | 0.101 | 4949 |
|  |  |  |  |  | 4916.66 |
|  | 10-1 | 1.684 | 1.786 | 0.102 | 4998 |
|  | 10-2 | 1.82 | 1.918 | 0.098 | 4802 |
|  | 10-3 | 1.721 | 1.817 | 0.096 | 4704 |
|  |  |  |  |  | 4834.666667 |
|  |  |  |  |  |  |

|  |  | 1 | 2 | 3 | average |  |
| --- | --- | --- | --- | --- | --- | --- |
|  | 8-0 | 4949 | 4949 | 5096 | 4998 |  |
|  | 8-1 | 6027 | 6566 | 6223 | 6272 |  |
|  | 8-3 | 10192 | 11907 | 11613 | 11237.33 |  |
|  | 8-5 | 11025 | 11172 | 11025 | 11074 |  |
|  | 8-7 | 4900 | 4900.98 | 4949 | 4916.66 |  |
|  |  |  |  |  |  |  |
|  | 10-0 | 4214 | 4116 | 4165 | 4165 |  |
|  | 10-1 | 5929 | 5341 | 5341 | 5537 |  |
|  | 10-3 | 9849 | 9947 | 9212 | 9669.33 |  |
|  | 10-5 | 9604 | 9849 | 10094 | 9849 |  |
|  | 10-7 | 4998 | 4802 | 4704 | 4834.666667 |  |
|  |  |  |  |  |  |  |
| The letter marks indicate the result |  |  |  | treat |  | SE |
| treat |  | 5%significant levels | 5%显著水平 | 8-0 |  | 49 |
| 8--3 |  | 11237.3333 | a | 8-1 |  | 157.513 |
| 8--5 |  | 11074 | a | 8-3 |  | 529.5125 |
| 10--5 |  | 9849 | b | 8-5 |  | 49 |
| 10--3 |  | 9669.333 | b | 8-7 |  | 16.1725 |
| 8--1 |  | 6272 | c | 10-0 |  | 28.2902 |
| 10--1 |  | 5537 | d | 10-1 |  | 196 |
| 8-0 |  | 4998 | de | 10-3 |  | 230.41 |
| 8--7 |  | 4916.6602 | de | 10-5 |  | 141.4508 |
| 10--7 |  | 4834.6667 | e | 10-7 |  | 86.4279 |
| 10-0 |  | 4165 | f |  |  |  |
|  |  |  |  |  |  |  |
|  | 0 | 1 | 3 | 5 | 7 |  |
| Baimi 8 | 4998 | 6272 | 11237.33333 | 11074 | 4916.66 |  |
| Baimi 10 | 4165 | 5537 | 9669.333333 | 9849 | 4834.666667 |  |
