# Supplementary material for: Evaluation of waterlogging tolerance and responses of protective enzymes to waterlogging stress in pumpkin
Source: PeerJ. 2023 Apr 21;11:e15177. doi: 10.7717/peerj.15177 (PMC10124548; doi:10.7717/peerj.15177)
Supplement: Supplemental Information 3 [file peerj-11-15177-s003.docx]

| treat day | variety | | A1 | A2 | △A | | CAT | | |
| --- | --- | --- | --- | --- | --- | --- | --- | --- | --- |
|  |  | |  |  | △A=A1-A2 | | CAT=459×△A/0.1 | | |
| 0d | 8-1 | | 1.225 | 1.165 | 0.06 | | 275.4 | | |
|  | 8-2 | | 1.186 | 1.13 | 0.056 | | 257.04 | | |
|  | 8-3 | | 1.071 | 1.024 | 0.047 | | 215.73 | | |
|  |  | |  |  |  | | 249.39 | | |
|  | 10-1 | | 0.777 | 0.731 | 0.046 | | 211.14 | | |
|  | 10-2 | | 1.224 | 1.168 | 0.056 | | 257.04 | | |
|  | 10-3 | | 1.225 | 1.168 | 0.057 | | 261.63 | | |
|  |  | |  |  |  | | 243.27 | | |
| 1d | 8-1 | | 1.563 | 1.397 | 0.166 | | 761.94 | | |
|  | 8-2 | | 1.613 | 1.453 | 0.16 | | 734.4 | | |
|  | 8-3 | | 1.586 | 1.423 | 0.163 | | 748.17 | | |
|  |  | |  |  |  | | 748.17 | | |
|  | 10-1 | | 1.414 | 1.219 | 0.195 | | 895.05 | | |
|  | 10-2 | | 1.882 | 1.702 | 0.18 | | 826.2 | | |
|  | 10-3 | | 1.647 | 1.4605 | 0.1865 | | 856.035 | | |
|  |  | |  |  |  | | 859.095 | | |
| 3d | 8-1 | | 2.099 | 1.935 | 0.164 | | 752.76 | | |
|  | 8-2 | | 2.101 | 1.934 | 0.167 | | 766.53 | | |
|  | 8-3 | | 2.118 | 1.953 | 0.165 | | 757.35 | | |
|  |  | |  |  |  | | 758.88 | | |
|  | 10-1 | | 1.705 | 1.486 | 0.219 | | 1005.21 | | |
|  | 10-2 | | 1.765 | 1.543 | 0.222 | | 1018.98 | | |
|  | 10-3 | | 1.794 | 1.568 | 0.226 | | 1037.34 | | |
|  |  | |  |  |  | | 1020.51 | | |
| 5d | 8-1 | | 1.331 | 1.174 | 0.157 | | 720.63 | | |
|  | 8-2 | | 1.327 | 1.184 | 0.143 | | 656.37 | | |
|  | 8-3 | | 1.318 | 1.179 | 0.139 | | 638.01 | | |
|  |  | |  |  |  | | 671.67 | | |
|  | 10-1 | | 1.214 | 1.014 | 0.2 | | 918 | | |
|  | 10-2 | | 1.256 | 1.021 | 0.235 | | 1078.65 | | |
|  | 10-3 | | 0.833 | 0.619 | 0.214 | | 982.26 | | |
|  |  | |  |  |  | | 992.97 | | |
| 7d | 8-1 | | 0.397 | 0.365 | 0.032 | | 146.88 | | |
|  | 8-2 | | 1.69 | 1.631 | 0.059 | | 270.81 | | |
|  | 8-3 | | 0.776 | 0.743 | 0.033 | | 151.47 | | |
|  |  | |  |  |  | | 189.72 | | |
|  | 10-1 | | 0.39 | 0.323 | 0.067 | | 307.53 | | |
|  | 10-2 | | 0.473 | 0.424 | 0.049 | | 224.91 | | |
|  | 10-3 | | 1.134 | 1.046 | 0.088 | | 403.92 | | |
|  |  | |  |  |  | | 312.12 | | |
|  |  | |  |  |  | |  | | |
|  | | 1 | 2 | 3 | | average | |  |  |
| 8-0 | | 275.4 | 257.04 | 215.73 | | 249.39 | |  |  |
| 8-1 | | 761.94 | 734.4 | 748.17 | | 748.17 | |  |  |
| 8-3 | | 752.76 | 766.53 | 757.35 | | 758.88 | |  |  |
| 8-5 | | 720.63 | 656.37 | 638.01 | | 671.67 | |  |  |
| 8-7 | | 146.88 | 270.81 | 151.47 | | 189.72 | |  |  |
|  | |  |  |  | |  | |  |  |
| 10-0 | | 211.14 | 257.04 | 261.63 | | 243.27 | |  |  |
| 10-1 | | 895.05 | 826.2 | 856.035 | | 859.095 | |  |  |
| 10-3 | | 1005.21 | 1018.98 | 1037.34 | | 1020.51 | |  |  |
| 10-5 | | 918 | 1078.65 | 982.26 | | 992.97 | |  |  |
| 10-7 | | 307.53 | 224.91 | 403.92 | | 312.12 | |  |  |
|  | |  |  |  | |  | |  |  |
|  | |  |  |  | |  | |  |  |
| The letter marks indicate the result | |  |  |  | |  | |  |  |
| treat | | average | 5%significant levels | treat | | average | | SE |  |
| 10-3d | | 1020.51 | a | 8-0 | | 249.39 | | 17.6448 |  |
| 10-5d | | 992.97 | a | 8-1 | | 748.17 | | 7.9501 |  |
| 10-1d | | 859.095 | b | 8-3 | | 839.97 | | 13.0723 |  |
| 8-1d | | 748.17 | c | 8-5 | | 671.67 | | 25.0472 |  |
| 8-3d | | 742.05 | c | 8-7 | | 189.72 | | 40.5666 |  |
| 8-5d | | 671.67 | c | 10-0 | | 243.27 | | 16.1196 |  |
| 10-7d | | 312.12 | d | 10-1 | | 767.295 | | 19.9341 |  |
| 8-0 | | 249.39 | de | 10-3 | | 974.61 | | 9.3066 |  |
| 10-0 | | 243.27 | de | 10-5 | | 934.83 | | 46.6838 |  |
| 8-7d | | 189.72 | e | 10-7 | | 312.12 | | 51.7267 |  |
|  | |  |  |  | |  | |  |  |
|  | |  |  |  | |  | |  |  |
|  | | 0 | 1 | 3 | | 5 | | 7 |  |
| Baimi 8 | | 249.39 | 748.17 | 758.88 | | 671.67 | | 189.72 |  |
| Baimi 10 | | 243.27 | 859.095 | 1020.51 | | 934.83 | | 312.12 |  |
