# Supplementary material for: Evaluation of waterlogging tolerance and responses of protective enzymes to waterlogging stress in pumpkin
Source: PeerJ. 2023 Apr 21;11:e15177. doi: 10.7717/peerj.15177 (PMC10124548; doi:10.7717/peerj.15177)
Supplement: Supplemental Information 4 [file peerj-11-15177-s004.docx]

| treat day | variety | A1 | A2 | B1 | A3 | A4 | B2 | APX（U/g） |
| --- | --- | --- | --- | --- | --- | --- | --- | --- |
|  |  |  |  | B1=（A1-A2） |  |  | B2=（A3-A4） | （APX=1.79×（B1-B2）/0.1） |
| 0d | 8-1 | 0.5453 | 0.5287 | 0.0166 | 1.523 | 1.49 | 0.033 | 0.2936 |
|  | 8-2 | 0.5453 | 0.5287 | 0.0166 | 1.833 | 1.798 | 0.035 | 0.3294 |
|  | 8-3 | 0.5453 | 0.5287 | 0.0166 | 1.695 | 1.657 | 0.038 | 0.3831 |
|  |  |  |  |  |  |  |  | 0.3353 |
|  | 10-1 | 0.5453 | 0.5287 | 0.0166 | 0.739 | 0.704 | 0.035 | 0.3294 |
|  | 10-2 | 0.5453 | 0.5287 | 0.0166 | 1.707 | 1.669 | 0.038 | 0.3831 |
|  | 10-3 | 0.5453 | 0.5287 | 0.0166 | 2.001 | 1.96 | 0.041 | 0.4368 |
|  |  |  |  |  |  |  |  | 0.3831 |
| 1d | 8-1 | 0.5453 | 0.5287 | 0.0166 | 1.802 | 1.778 | 0.024 | 0.1325 |
|  | 8-2 | 0.5453 | 0.5287 | 0.0166 | 1.256 | 1.231 | 0.025 | 0.1504 |
|  | 8-3 | 0.5453 | 0.5287 | 0.0166 | 1.695 | 1.672 | 0.023 | 0.1146 |
|  |  |  |  |  |  |  |  | 0.1325 |
|  | 10-1 | 0.5453 | 0.5287 | 0.0166 | 1.622 | 1.59 | 0.032 | 0.2757 |
|  | 10-2 | 0.5453 | 0.5287 | 0.0166 | 1.66 | 1.627 | 0.033 | 0.2936 |
|  | 10-3 | 0.5453 | 0.5287 | 0.0166 | 1.615 | 1.586 | 0.029 | 0.2220 |
|  |  |  |  |  |  |  |  | 0.2637 |
| 3d | 8-1 | 0.5453 | 0.5287 | 0.0166 | 1.769 | 1.739 | 0.03 | 0.2399 |
|  | 8-2 | 0.5453 | 0.5287 | 0.0166 | 1.221 | 1.195 | 0.026 | 0.1683 |
|  | 8-3 | 0.5453 | 0.5287 | 0.0166 | 1.336 | 1.306 | 0.03 | 0.2399 |
|  |  |  |  |  |  |  |  | 0.2160 |
|  | 10-1 | 0.5453 | 0.5287 | 0.0166 | 1.351 | 1.321 | 0.03 | 0.2399 |
|  | 10-2 | 0.5453 | 0.5287 | 0.0166 | 1.389 | 1.357 | 0.032 | 0.2757 |
|  | 10-3 | 0.5453 | 0.5287 | 0.0166 | 1.351 | 1.32 | 0.031 | 0.2578 |
|  |  |  |  |  |  |  |  | 0.2577600 |
| 5d | 8-1 | 0.5453 | 0.5287 | 0.0166 | 0.229 | 0.2 | 0.029 | 0.2220 |
|  | 8-2 | 0.5453 | 0.5287 | 0.0166 | 0.753 | 0.728 | 0.025 | 0.1504 |
|  | 8-3 | 0.5453 | 0.5287 | 0.0166 | 0.524 | 0.498 | 0.026 | 0.1683 |
|  |  |  |  |  |  |  |  | 0.1802 |
|  | 10-1 | 0.5453 | 0.5287 | 0.0166 | 0.6 | 0.562 | 0.038 | 0.3831 |
|  | 10-2 | 0.5453 | 0.5287 | 0.0166 | 0.628 | 0.591 | 0.037 | 0.3652 |
|  | 10-3 | 0.5453 | 0.5287 | 0.0166 | 0.54 | 0.51 | 0.03 | 0.2399 |
|  |  |  |  |  |  |  |  | 0.3294 |
| 7d | 8-1 | 0.5453 | 0.5287 | 0.0166 | 1.018 | 0.995 | 0.023 | 0.1146 |
|  | 8-2 | 0.5453 | 0.5287 | 0.0166 | 0.98 | 0.958 | 0.022 | 0.0967 |
|  | 8-3 | 0.5453 | 0.5287 | 0.0166 | 0.757 | 0.739 | 0.018 | 0.0251 |
|  |  |  |  |  |  |  |  | 0.0788 |
|  | 10-1 | 0.5453 | 0.5287 | 0.0166 | 0.548 | 0.521 | 0.027 | 0.1862 |
|  | 10-2 | 0.5453 | 0.5287 | 0.0166 | 0.642 | 0.621 | 0.021 | 0.0788 |
|  | 10-3 | 0.5453 | 0.5287 | 0.0166 | 0.595 | 0.571 | 0.024 | 0.1325 |
|  |  |  |  |  |  |  |  | 0.1325 |

|  | 8-1 | 8-2 | 8-3 |  |  |  |
| --- | --- | --- | --- | --- | --- | --- |
| 0d | 0.29356 | 0.32936 | 0.38306 | 0.335326667 |  |  |
| 1d | 0.13246 | 0.15036 | 0.11456 | 0.13246 |  |  |
| 3d | 0.23986 | 0.16826 | 0.23986 | 0.215993333 |  |  |
| 5d | 0.22196 | 0.15036 | 0.16826 | 0.180193333 |  |  |
| 7d | 0.11456 | 0.09666 | 0.02506 | 0.07876 |  |  |
|  | 10-1 | 10--2 | 10-3 |  |  |  |
| 0d | 0.32936 | 0.38306 | 0.43676 | 0.38306 |  |  |
| 1d | 0.27566 | 0.29356 | 0.22196 | 0.263726667 |  |  |
| 3d | 0.23986 | 0.27566 | 0.25776 | 0.25776 |  |  |
| 5d | 0.38306 | 0.36516 | 0.23986 | 0.32936 |  |  |
| 7d | 0.18616 | 0.07876 | 0.13246 | 0.13246 |  |  |
|  |  |  |  |  |  |  |
|  |  |  |  | The letter marks indicate the result |  |  |
| treat |  | SE |  | treat | 5%significant levels |  |
| 8-0 |  | 0.045 |  | 10-0 | a |  |
| 8-1 |  | 0.0179 |  | 8-0 | ab |  |
| 8-3 |  | 0.0413 |  | 10-5 | ab |  |
| 8-5 |  | 0.0373 |  | 10-1 | bc |  |
| 8-7 |  | 0.0474 |  | 10-3 | bc |  |
| 10-0 |  | 0.0537 |  | 8-3 | cd |  |
| 10-1 |  | 0.0373 |  | 8-5 | cd |  |
| 10-3 |  | 0.0179 |  | 8-1 | de |  |
| 10-5 |  | 0.078 |  | 10-7 | de |  |
| 10-7 |  | 0.0537 |  | 8-7 | e |  |
|  |  |  |  |  |  |  |
|  |  | 0 | 1 | 3 | 5 | 7 |
|  | Baimi 8 | 0.335326667 | 0.13246 | 0.215993333 | 0.180193333 | 0.07876 |
|  | Baimi 10 | 0.38306 | 0.263726667 | 0.25776 | 0.32936 | 0.13246 |
|  |  |  |  |  |  |  |
