# Supplementary material for: Evaluation of waterlogging tolerance and responses of protective enzymes to waterlogging stress in pumpkin
Source: PeerJ. 2023 Apr 21;11:e15177. doi: 10.7717/peerj.15177 (PMC10124548; doi:10.7717/peerj.15177)
Supplement: Supplemental Information 5 [file peerj-11-15177-s005.docx]

|  | |  | | OD450CK | | OD532CK | | OD600CK | | OD450 | | △OD450 | OD532 | △OD532 | OD600 | △OD600 | MDA |
| --- | --- | --- | --- | --- | --- | --- | --- | --- | --- | --- | --- | --- | --- | --- | --- | --- | --- |
| treat day | | variety | | 空白450nm | | 空白532nm | | 空白600nm | | 测定450nm | | △450 | 测定532nm | △532 | 测定600nm | △600 | nmol/g |
|  | |  | |  | |  | |  | |  | | △OD450=OD450-OD450CK |  | △OD450=OD450-OD450CK |  | △OD450=OD450-OD450CK | MDA=5×{12.9×(△OD532-△OD600)-2.58×△OD450}/0.1 |
| 0d | | 8-1 | | 0.045 | | 0.058 | | 0.03 | | 0.148 | | 0.103 | 0.271 | 0.213 | 0.061 | 0.031 | 104.103 |
|  | | 8-2 | | 0.045 | | 0.058 | | 0.03 | | 0.147 | | 0.102 | 0.232 | 0.174 | 0.059 | 0.029 | 80.367 |
|  | | 8-3 | | 0.045 | | 0.058 | | 0.03 | | 0.138 | | 0.1025 | 0.393 | 0.1935 | 0.065 | 0.065 | 69.66 |
|  | |  | |  | |  | |  | |  | |  |  |  |  |  | 92.235 |
|  | | 10-1 | | 0.045 | | 0.058 | | 0.03 | | 0.134 | | 0.089 | 0.28 | 0.222 | 0.101 | 0.071 | 85.914 |
|  | | 10-2 | | 0.045 | | 0.058 | | 0.03 | | 0.128 | | 0.083 | 0.278 | 0.22 | 0.083 | 0.053 | 97.008 |
|  | | 10-3 | | 0.045 | | 0.058 | | 0.03 | | 0.136 | | 0.091 | 0.286 | 0.228 | 0.079 | 0.049 | 103.716 |
|  | |  | |  | |  | |  | |  | |  |  |  |  |  | 95.546 |
| 1d | | 8-1 | | 0.045 | | 0.058 | | 0.03 | | 0.211 | | 0.166 | 0.321 | 0.263 | 0.089 | 0.059 | 110.166 |
|  | | 8-2 | | 0.045 | | 0.058 | | 0.03 | | 0.215 | | 0.17 | 0.337 | 0.279 | 0.088 | 0.058 | 120.615 |
|  | | 8-3 | | 0.045 | | 0.058 | | 0.03 | | 0.211 | | 0.166 | 0.331 | 0.273 | 0.087 | 0.057 | 117.906 |
|  | |  | |  | |  | |  | |  | |  |  |  |  |  | 116.229 |
|  | | 10-1 | | 0.045 | | 0.058 | | 0.03 | | 0.21 | | 0.165 | 0.306 | 0.248 | 0.095 | 0.065 | 96.75 |
|  | | 10-2 | | 0.045 | | 0.058 | | 0.03 | | 0.189 | | 0.144 | 0.298 | 0.24 | 0.1 | 0.07 | 91.074 |
|  | | 10-3 | | 0.045 | | 0.058 | | 0.03 | | 0.259 | | 0.214 | 0.343 | 0.285 | 0.098 | 0.068 | 112.359 |
|  | |  | |  | |  | |  | |  | |  |  |  |  |  | 100.061 |
| 3d | | 8-1 | | 0.045 | | 0.058 | | 0.03 | | 0.211 | | 0.166 | 0.351 | 0.293 | 0.043 | 0.013 | 159.186 |
|  | | 8-2 | | 0.045 | | 0.058 | | 0.03 | | 0.248 | | 0.203 | 0.408 | 0.35 | 0.038 | 0.008 | 194.403 |
|  | | 8-3 | | 0.045 | | 0.058 | | 0.03 | | 0.209 | | 0.1845 | 0.335 | 0.3215 | 0.01 | 0.01 | 177.117 |
|  | |  | |  | |  | |  | |  | |  |  |  |  |  | 176.7945 |
|  | | 10-1 | | 0.045 | | 0.058 | | 0.03 | | 0.198 | | 0.153 | 0.267 | 0.209 | 0.035 | 0.005 | 111.843 |
|  | | 10-2 | | 0.045 | | 0.058 | | 0.03 | | 0.285 | | 0.24 | 0.308 | 0.25 | 0.068 | 0.038 | 105.78 |
|  | | 10-3 | | 0.045 | | 0.058 | | 0.03 | | 0.239 | | 0.1965 | 0.284 | 0.2295 | 0.046 | 0.016 | 112.359 |
|  | |  | |  | |  | |  | |  | |  |  |  |  |  | 109.994 |
| 5d | | 8-1 | | 0.045 | | 0.058 | | 0.03 | | 0.329 | | 0.284 | 0.522 | 0.464 | 0.075 | 0.045 | 233.619 |
|  | | 8-2 | | 0.045 | | 0.058 | | 0.03 | | 0.253 | | 0.208 | 0.469 | 0.411 | 0.076 | 0.046 | 208.593 |
|  | | 8-3 | | 0.045 | | 0.058 | | 0.03 | | 0.283 | | 0.238 | 0.378 | 0.32 | 0.095 | 0.065 | 133.773 |
|  | |  | |  | |  | |  | |  | |  |  |  |  |  | 191.995 |
|  | | 10-1 | | 0.045 | | 0.058 | | 0.03 | | 0.16 | | 0.115 | 0.282 | 0.224 | 0.03 | 0 | 129.645 |
|  | | 10-2 | | 0.045 | | 0.058 | | 0.03 | | 0.505 | | 0.46 | 0.621 | 0.563 | 0.265 | 0.235 | 152.22 |
|  | | 10-3 | | 0.045 | | 0.058 | | 0.03 | | 0.252 | | 0.2875 | 0.355 | 0.297 | 0.035 | 0.005 | 151.2525 |
|  | |  | |  | |  | |  | |  | |  |  |  |  |  | 151.73625 |
| 7d | | 8-1 | | 0.045 | | 0.058 | | 0.03 | | 0.241 | | 0.196 | 0.397 | 0.339 | 0.038 | 0.008 | 188.211 |
|  | | 8-2 | | 0.045 | | 0.058 | | 0.03 | | 0.51 | | 0.465 | 0.583 | 0.525 | 0.165 | 0.135 | 191.565 |
|  | | 8-3 | | 0.045 | | 0.058 | | 0.03 | | 0.349 | | 0.304 | 0.471 | 0.413 | 0.075 | 0.045 | 198.144 |
|  | |  | |  | |  | |  | |  | |  |  |  |  |  | 192.64 |
|  | | 10-1 | | 0.045 | | 0.058 | | 0.03 | | 0.161 | | 0.116 | 0.342 | 0.284 | 0.045 | 0.015 | 158.541 |
|  | | 10-2 | | 0.045 | | 0.058 | | 0.03 | | 0.185 | | 0.14 | 0.37 | 0.312 | 0.031 | 0.001 | 182.535 |
|  | | 10-3 | | 0.045 | | 0.058 | | 0.03 | | 0.149 | | 0.104 | 0.272 | 0.214 | 0.031 | 0.001 | 123.969 |
|  | |  | |  | |  | |  | |  | |  |  |  |  |  | 155.015 |
|  | |  | |  | |  | |  | |  | |  |  |  |  |  |  |
|  | |  | |  | |  | |  | |  | |  |  |  |  |  |  |
|  | |  | |  | |  | |  | |  | |  |  |  |  |  |  |
|  | 8-1 | | 8-2 | | 8-3 | | average | |  | |  |  |  |  |  |  |  |
| 0d | 104.103 | | 80.367 | | 69.66 | | 84.71 | |  | |  |  |  |  |  |  |  |
| 1d | 110.166 | | 120.615 | | 117.906 | | 116.229 | |  | |  |  |  |  |  |  |  |
| 3d | 159.186 | | 194.403 | | 177.117 | | 176.7945 | |  | |  |  |  |  |  |  |  |
| 5d | 233.619 | | 208.593 | | 133.773 | | 191.995 | |  | |  |  |  |  |  |  |  |
| 7d | 188.211 | | 191.565 | | 198.144 | | 192.64 | |  | |  |  |  |  |  |  |  |
|  | 10-1 | | 10--2 | | 10-3 | |  | |  | |  |  |  |  |  |  |  |
| 0d | 85.914 | | 97.008 | | 103.716 | | 95.546 | |  | |  |  |  |  |  |  |  |
| 1d | 96.75 | | 91.074 | | 112.359 | | 100.061 | |  | |  |  |  |  |  |  |  |
| 3d | 111.843 | | 105.78 | | 112.359 | | 109.994 | |  | |  |  |  |  |  |  |  |
| 5d | 129.645 | | 152.22 | | 151.2525 | | 151.73625 | |  | |  |  |  |  |  |  |  |
| 7d | 158.541 | | 182.535 | | 123.969 | | 155.015 | |  | |  |  |  |  |  |  |  |
|  |  | |  | |  | |  | |  | |  |  |  |  |  |  |  |
|  |  | |  | |  | |  | |  | |  |  |  |  |  |  |  |
|  |  | |  | |  | |  | |  | |  |  |  |  |  |  |  |
| The letter marks indicate the result |  | |  | |  | |  | |  | |  |  |  |  |  |  |  |
| treat | 5%significant levels | |  | |  | |  | |  | |  |  |  |  |  |  |  |
| 8-7 | a | |  | | treat | | average | | SE | |  |  |  |  |  |  |  |
| 8-5 | a | |  | | 8-0 | | 84.71 | | 17.6274 | |  |  |  |  |  |  |  |
| 8-3 | ab | |  | | 8-1 | | 116.229 | | 5.4226 | |  |  |  |  |  |  |  |
| 10-7 | ab | |  | | 8-3 | | 176.902 | | 17.6095 | |  |  |  |  |  |  |  |
| 10-5 | bc | |  | | 8-5 | | 191.995 | | 31.9512 | |  |  |  |  |  |  |  |
| 8-1 | cd | |  | | 8-7 | | 192.64 | | 5.053 | |  |  |  |  |  |  |  |
| 10-3 | cd | |  | | 10-0 | | 95.546 | | 8.9906 | |  |  |  |  |  |  |  |
| 10-1 | d | |  | | 10-1 | | 100.061 | | 11.022 | |  |  |  |  |  |  |  |
| 10-0 | d | |  | | 10-3 | | 109.994 | | 3.6585 | |  |  |  |  |  |  |  |
| 8-0 | d | |  | | 10-5 | | 144.3725 | | 12.7636 | |  |  |  |  |  |  |  |
|  |  | |  | | 10-7 | | 155.015 | | 29.4418 | |  |  |  |  |  |  |  |
|  | 0 | | 1 | | 3 | | 5 | | 7 | |  |  |  |  |  |  |  |
| Baimi 8 | 84.71 | | 116.229 | | 176.7945 | | 191.995 | | 192.64 | |  |  |  |  |  |  |  |
| Baimi 10 | 95.546 | | 100.061 | | 109.994 | | 151.73625 | | 155.015 | |  |  |  |  |  |  |  |
|  |  | |  | |  | |  | |  | |  |  |  |  |  |  |  |
