# Supplementary material for: Evaluation of waterlogging tolerance and responses of protective enzymes to waterlogging stress in pumpkin
Source: PeerJ. 2023 Apr 21;11:e15177. doi: 10.7717/peerj.15177 (PMC10124548; doi:10.7717/peerj.15177)
Supplement: Supplemental Information 6 [file peerj-11-15177-s006.docx]

| treat day | variety | OD | A(μg/mL) | PRO(μg/g) |
| --- | --- | --- | --- | --- |
|  |  | 520nm OD值 | 测定液中脯氨酸含量 | 单位鲜质量样品脯氨酸含量 |
|  |  |  | A=(OD-0.0927)/0.0436 | PRO =A/0.1 |
| 0d | 8-1 | 0.554 | 10.58027523 | 105.8027523 |
|  | 8-2 | 0.616 | 12.00229358 | 120.0229358 |
|  | 8-3 | 0.569 | 10.92431193 | 109.2431193 |
|  |  |  |  | 111.6896024 |
|  | 10-1 | 0.363 | 6.199541284 | 61.99541284 |
|  | 10-2 | 0.222 | 2.96559633 | 29.6559633 |
|  | 10-3 | 0.307 | 4.915137615 | 49.15137615 |
|  |  |  |  | 46.93425076 |
| 1d | 8-1 | 0.486 | 9.020642202 | 90.20642202 |
|  | 8-2 | 0.498 | 9.29587156 | 92.9587156 |
|  | 8-3 | 0.515 | 9.685779817 | 96.85779817 |
|  |  |  |  | 93.34097859 |
|  | 10-1 | 0.492 | 9.158256881 | 91.58256881 |
|  | 10-2 | 0.514 | 9.662844037 | 96.62844037 |
|  | 10-3 | 0.506 | 9.479357798 | 94.79357798 |
|  |  |  |  | 94.33486239 |
| 3d | 8-1 | 0.934 | 19.29587156 | 192.9587156 |
|  | 8-2 | 0.891 | 18.30963303 | 183.0963303 |
|  | 8-3 | 0.784 | 15.85550459 | 158.5550459 |
|  |  |  |  | 178.2033639 |
|  | 10-1 | 1.063 | 22.25458716 | 222.5458716 |
|  | 10-2 | 0.986 | 20.48853211 | 204.8853211 |
|  | 10-3 | 1.25 | 26.54357798 | 265.4357798 |
|  |  |  |  | 230.9556575 |
| 5d | 8-1 | 0.492 | 9.158256881 | 91.58256881 |
|  | 8-2 | 0.321 | 5.236238532 | 52.36238532 |
|  | 8-3 | 0.351 | 5.924311927 | 59.24311927 |
|  |  |  |  | 67.7293578 |
|  | 10-1 | 0.386 | 6.72706422 | 67.2706422 |
|  | 10-2 | 0.313 | 5.052752294 | 50.52752294 |
|  | 10-3 | 0.492 | 9.158256881 | 91.58256881 |
|  |  |  |  | 69.79357798 |
| 7d | 8-1 | 0.406 | 7.185779817 | 71.85779817 |
|  | 8-2 | 0.354 | 5.993119266 | 59.93119266 |
|  | 8-3 | 0.402 | 7.094036697 | 70.94036697 |
|  |  |  |  | 67.5764526 |
|  | 10-1 | 0.316 | 5.121559633 | 51.21559633 |
|  | 10-2 | 0.299 | 4.731651376 | 47.31651376 |
|  | 10-3 | 0.38 | 6.589449541 | 65.89449541 |
|  |  |  |  | 54.8088685 |
|  |  |  |  |  |

|  | 8-1 | 8-2 | 8-3 | average |  |
| --- | --- | --- | --- | --- | --- |
| 0d | 105.8027523 | 120.0229358 | 109.2431193 | 111.6896024 |  |
| 1d | 90.20642202 | 92.9587156 | 96.85779817 | 93.34097859 |  |
| 3d | 192.9587156 | 183.0963303 | 158.5550459 | 178.2033639 |  |
| 5d | 91.58256881 | 52.36238532 | 59.24311927 | 67.7293578 |  |
| 7d | 71.85779817 | 59.93119266 | 70.94036697 | 67.5764526 |  |
|  | 10-1 | 10--2 | 10-3 |  |  |
| 0d | 61.99541284 | 29.6559633 | 49.15137615 | 46.93425076 |  |
| 1d | 91.58256881 | 96.62844037 | 94.79357798 | 94.33486239 |  |
| 3d | 222.5458716 | 204.8853211 | 265.4357798 | 230.9556575 |  |
| 5d | 67.2706422 | 50.52752294 | 91.58256881 | 69.79357798 |  |
| 7d | 51.21559633 | 47.31651376 | 65.89449541 | 54.8088685 |  |
|  |  |  | The letter marks indicate the result |  |  |
| treat | average | SE | treat | average | 5%significant levels |
| 8-0d | 111.6896 | 4.2834 | 10-3d | 230.9557 | a |
| 8-1 | 93.341 | 1.9296 | 8-3d | 178.2034 | b |
| 8-3 | 178.2034 | 10.2284 | 8-0 | 111.6896 | c |
| 8-5 | 67.7294 | 12.0909 | 10-1d | 94.3349 | cd |
| 8-7 | 67.5765 | 3.8318 | 8-1d | 93.341 | cd |
| 10-0 | 46.9343 | 9.4012 | 10-5d | 69.7936 | de |
| 10-1 | 94.3349 | 1.4746 | 8-5d | 67.7294 | de |
| 10-3 | 230.9557 | 17.9781 | 8-7d | 67.5765 | de |
| 10-5 | 69.7936 | 11.9185 | 10-7d | 54.8089 | e |
| 10-7 | 54.8089 | 5.6559 | 10-0 | 46.9342 | e |
|  |  |  |  |  |  |
|  | 0 | 1 | 3 | 5 | 7 |
| Baimi 8 | 111.6896024 | 93.34097859 | 178.2033639 | 67.7293578 | 67.5764526 |
| Baimi 10 | 46.93425076 | 94.33486239 | 230.9556575 | 69.79357798 | 54.8088685 |
