# Supplementary material for: Evaluation of waterlogging tolerance and responses of protective enzymes to waterlogging stress in pumpkin
Source: PeerJ. 2023 Apr 21;11:e15177. doi: 10.7717/peerj.15177 (PMC10124548; doi:10.7717/peerj.15177)
Supplement: Supplemental Information 7 [file peerj-11-15177-s007.docx]

| treat day | variety | blankA1 | blankA2 | blank | measureA3 | measureA4 | measure | ADH（U/g） |
| --- | --- | --- | --- | --- | --- | --- | --- | --- |
|  |  | A1 | A2 | △A12=A1-A2 | A3 | A4 | △A34=A3-A4 | ADH=1.61*(A34-A12/0.1 |
| 0d | 8-1 | 0.03 | 0.028 | 0.002 | 0.065 | 0.053 | 0.012 | 0.161 |
|  | 8-2 | 0.03 | 0.028 | 0.002 | 0.067 | 0.056 | 0.011 | 0.1449 |
|  | 8-3 | 0.03 | 0.028 | 0.002 | 0.063 | 0.052 | 0.011 | 0.1449 |
|  |  |  |  |  |  |  |  | 0.150266667 |
|  | 10-1 | 0.03 | 0.028 | 0.002 | 0.132 | 0.123 | 0.009 | 0.1127 |
|  | 10-2 | 0.03 | 0.028 | 0.002 | 0.058 | 0.048 | 0.01 | 0.1288 |
|  | 10-3 | 0.03 | 0.028 | 0.002 | 0.029 | 0.019 | 0.01 | 0.1288 |
|  |  |  |  |  |  |  |  | 0.123433333 |
| 1d | 8-1 | 0.03 | 0.028 | 0.002 | 0.049 | 0.043 | 0.006 | 0.0644 |
|  | 8-2 | 0.03 | 0.028 | 0.002 | 0.042 | 0.034 | 0.008 | 0.0966 |
|  | 8-3 | 0.03 | 0.028 | 0.002 | 0.024 | 0.017 | 0.007 | 0.0805 |
|  |  |  |  |  |  |  |  | 0.0805 |
|  | 10-1 | 0.03 | 0.028 | 0.002 | 0.056 | 0.047 | 0.009 | 0.1127 |
|  | 10-2 | 0.03 | 0.028 | 0.002 | 0.049 | 0.042 | 0.007 | 0.0805 |
|  | 10-3 | 0.03 | 0.028 | 0.002 | 0.067 | 0.06 | 0.007 | 0.0805 |
|  |  |  |  |  |  |  |  | 0.091233333 |
| 3d | 8-1 | 0.03 | 0.028 | 0.002 | 0.102 | 0.092 | 0.01 | 0.1288 |
|  | 8-2 | 0.03 | 0.028 | 0.002 | 0.086 | 0.077 | 0.009 | 0.1127 |
|  | 8-3 | 0.03 | 0.028 | 0.002 | 0.094 | 0.087 | 0.007 | 0.0805 |
|  |  |  |  |  |  |  |  | 0.107333333 |
|  | 10-1 | 0.03 | 0.028 | 0.002 | 0.161 | 0.151 | 0.01 | 0.1288 |
|  | 10-2 | 0.03 | 0.028 | 0.002 | 0.063 | 0.051 | 0.012 | 0.161 |
|  | 10-3 | 0.03 | 0.028 | 0.002 | 0.096 | 0.09 | 0.006 | 0.0644 |
|  |  |  |  |  |  |  |  | 0.118066667 |
| 5d | 8-1 | 0.03 | 0.028 | 0.002 | 0.099 | 0.09 | 0.009 | 0.1127 |
|  | 8-2 | 0.03 | 0.028 | 0.002 | 0.078 | 0.066 | 0.012 | 0.161 |
|  | 8-3 | 0.03 | 0.028 | 0.002 | 0.089 | 0.083 | 0.0105 | 0.13685 |
|  |  |  |  |  |  |  |  | 0.13685 |
|  | 10-1 | 0.03 | 0.028 | 0.002 | 0.075 | 0.066 | 0.009 | 0.1127 |
|  | 10-2 | 0.03 | 0.028 | 0.002 | 0.052 | 0.039 | 0.013 | 0.1771 |
|  | 10-3 | 0.03 | 0.028 | 0.002 | 0.117 | 0.106 | 0.011 | 0.1449 |
|  |  |  |  |  |  |  |  | 0.1449 |
| 7d | 8-1 | 0.03 | 0.028 | 0.002 | 0.08 | 0.074 | 0.006 | 0.0644 |
|  | 8-2 | 0.03 | 0.028 | 0.002 | 0.056 | 0.052 | 0.004 | 0.0322 |
|  | 8-3 | 0.03 | 0.028 | 0.002 | 0.054 | 0.049 | 0.005 | 0.0483 |
|  |  |  |  |  |  |  |  | 0.0483 |
|  | 10-1 | 0.03 | 0.028 | 0.002 | 0.079 | 0.072 | 0.007 | 0.0805 |
|  | 10-2 | 0.03 | 0.028 | 0.002 | 0.088 | 0.08 | 0.008 | 0.0966 |
|  | 10-3 | 0.03 | 0.028 | 0.002 | 0.08 | 0.076 | 0.004 | 0.0322 |
|  |  |  |  |  |  |  |  | 0.08855 |

|  | 1 | 2 | 3 | average |  |  |
| --- | --- | --- | --- | --- | --- | --- |
| 8-0 | 0.161 | 0.1449 | 0.1449 | 0.150266667 |  |  |
| 8-1 | 0.0644 | 0.0966 | 0.0805 | 0.0805 |  |  |
| 8-3 | 0.1288 | 0.1127 | 0.0805 | 0.107333333 |  |  |
| 8-5 | 0.1127 | 0.161 | 0.13685 | 0.13685 |  |  |
| 8-7 | 0.0644 | 0.0322 | 0.0483 | 0.0483 |  |  |
|  |  |  |  |  |  |  |
| 10-0 | 0.1127 | 0.1288 | 0.1288 | 0.123433333 |  |  |
| 10-1 | 0.1127 | 0.0805 | 0.0805 | 0.091233333 |  |  |
| 10-3 | 0.1288 | 0.161 | 0.0644 | 0.118066667 |  |  |
| 10-5 | 0.1127 | 0.1771 | 0.1449 | 0.1449 |  |  |
| 10-7 | 0.0805 | 0.0966 | 0.0322 | 0.08855 |  |  |
|  |  |  |  |  |  |  |
|  |  |  | The letter marks indicate the result |  |  |  |
| treat | average | SE | treat | average | 5%significant levels |  |
| 8-0 | 0.1503 | 0.0054 | 8-0 | 0.1503 | a |  |
| 8-1 | 0.0805 | 0.0093 | 10-5 | 0.1449 | a |  |
| 8-3 | 0.1073 | 0.0142 | 8--5 | 0.1368 | ab |  |
| 8-5 | 0.1368 | 0.0139 | 10-0 | 0.1234 | abc |  |
| 8-7 | 0.0483 | 0.0093 | 10--3 | 0.1181 | abcd |  |
| 10-0 | 0.1234 | 0.0054 | 8--3 | 0.1073 | abcd |  |
| 10-1 | 0.0912 | 0.0107 | 10--1 | 0.0912 | bcde |  |
| 10-3 | 0.1181 | 0.0284 | 8--1 | 0.0805 | cde |  |
| 10-5 | 0.1449 | 0.0186 | 10--7 | 0.0698 | de |  |
| 10-7 | 0.0698 | 0.0193 | 8--7 | 0.0483 | e |  |
|  |  |  |  |  |  |  |
|  |  |  |  |  |  |  |
|  |  |  |  |  |  |  |
|  | 0 | 1 | 3 | 5 | 7 |  |
| Baimi 8 | 0.150266667 | 0.0805 | 0.107333333 | 0.13685 | 0.0483 |  |
| Baimi 10 | 0.123433333 | 0.091233333 | 0.118066667 | 0.1449 | 0.08855 |  |
