# Supplementary material for: Evaluation of waterlogging tolerance and responses of protective enzymes to waterlogging stress in pumpkin
Source: PeerJ. 2023 Apr 21;11:e15177. doi: 10.7717/peerj.15177 (PMC10124548; doi:10.7717/peerj.15177)
Supplement: Supplemental Information 8 [file peerj-11-15177-s008.docx]

| treat day | variety | blankA3 | blankA4 | A3-A4 | measureA1 | measureA2 | A1-A2 | △A | （U/g） |
| --- | --- | --- | --- | --- | --- | --- | --- | --- | --- |
|  |  | A3 | A4 | △A34=A3-A4 | A1 | A2 | △A12=A1-A2 | △A=△A12-△A34 | PDC=1.6*△A/0.1 |
| 0d | 8-1 | 0.618 | 0.616 | 0.002 | 0.405 | 0.184 | 0.221 | 0.219 | 3.504 |
|  | 8-2 | 0.618 | 0.616 | 0.002 | 0.413 | 0.294 | 0.119 | 0.117 | 1.872 |
|  | 8-3 | 0.618 | 0.616 | 0.002 | 0.353 | 0.235 | 0.118 | 0.116 | 1.856 |
|  |  |  |  |  |  |  |  |  | 2.410666667 |
|  | 10-1 | 0.618 | 0.616 | 0.002 | 0.37 | 0.223 | 0.147 | 0.145 | 2.32 |
|  | 10-2 | 0.618 | 0.616 | 0.002 | 0.432 | 0.269 | 0.163 | 0.161 | 2.576 |
|  | 10-3 | 0.618 | 0.616 | 0.002 | 0.439 | 0.34 | 0.099 | 0.097 | 1.552 |
|  |  |  |  |  |  |  |  |  | 2.149333333 |
| 1d | 8-1 | 0.618 | 0.616 | 0.002 | 0.529 | 0.458 | 0.071 | 0.069 | 1.104 |
|  | 8-2 | 0.618 | 0.616 | 0.002 | 0.473 | 0.406 | 0.067 | 0.065 | 1.04 |
|  | 8-3 | 0.618 | 0.616 | 0.002 | 0.339 | 0.242 | 0.097 | 0.095 | 1.52 |
|  |  |  |  |  |  |  |  |  | 1.221333333 |
|  | 10-1 | 0.618 | 0.616 | 0.002 | 0.338 | 0.092 | 0.246 | 0.244 | 3.904 |
|  | 10-2 | 0.618 | 0.616 | 0.002 | 0.27 | 0.016 | 0.254 | 0.252 | 4.032 |
|  | 10-3 | 0.618 | 0.616 | 0.002 | 0.244 | 0.044 | 0.2 | 0.198 | 3.168 |
|  |  |  |  |  |  |  |  |  | 3.701333333 |
| 3d | 8-1 | 0.618 | 0.616 | 0.002 | 0.643 | 0.571 | 0.072 | 0.07 | 1.12 |
|  | 8-2 | 0.618 | 0.616 | 0.002 | 0.621 | 0.545 | 0.076 | 0.074 | 1.184 |
|  | 8-3 | 0.618 | 0.616 | 0.002 | 0.59 | 0.545 | 0.045 | 0.043 | 0.688 |
|  |  |  |  |  |  |  |  |  | 0.997333333 |
|  | 10-1 | 0.618 | 0.616 | 0.002 | 0.616 | 0.565 | 0.051 | 0.049 | 0.784 |
|  | 10-2 | 0.618 | 0.616 | 0.002 | 0.454 | 0.4 | 0.054 | 0.052 | 0.832 |
|  | 10-3 | 0.618 | 0.616 | 0.002 | 0.567 | 0.489 | 0.078 | 0.076 | 1.216 |
|  |  |  |  |  |  |  |  |  | 0.944 |
| 5d | 8-1 | 0.618 | 0.616 | 0.002 | 0.539 | 0.385 | 0.154 | 0.152 | 2.432 |
|  | 8-2 | 0.618 | 0.616 | 0.002 | 0.583 | 0.543 | 0.04 | 0.038 | 0.608 |
|  | 8-3 | 0.618 | 0.616 | 0.002 | 0.611 | 0.486 | 0.125 | 0.123 | 1.968 |
|  |  |  |  |  |  |  |  |  | 1.669333333 |
|  | 10-1 | 0.618 | 0.616 | 0.002 | 0.549 | 0.416 | 0.133 | 0.131 | 2.096 |
|  | 10-2 | 0.618 | 0.616 | 0.002 | 0.634 | 0.479 | 0.155 | 0.153 | 2.448 |
|  | 10-3 | 0.618 | 0.616 | 0.002 | 0.549 | 0.4 | 0.149 | 0.147 | 2.352 |
|  |  |  |  |  |  |  |  |  | 2.298666667 |
| 7d | 8-1 | 0.618 | 0.616 | 0.002 | 0.616 | 0.526 | 0.09 | 0.088 | 1.408 |
|  | 8-2 | 0.618 | 0.616 | 0.002 | 0.573 | 0.475 | 0.098 | 0.096 | 1.536 |
|  | 8-3 | 0.618 | 0.616 | 0.002 | 0.5945 | 0.5005 | 0.094 | 0.092 | 1.472 |
|  |  |  |  |  |  |  |  |  | 1.472 |
|  | 10-1 | 0.618 | 0.616 | 0.002 | 0.622 | 0.582 | 0.04 | 0.038 | 0.608 |
|  | 10-2 | 0.618 | 0.616 | 0.002 | 0.628 | 0.586 | 0.042 | 0.04 | 0.64 |
|  | 10-3 | 0.618 | 0.616 | 0.002 | 0.564 | 0.375 | 0.189 | 0.187 | 2.992 |
|  |  |  |  |  |  |  |  |  | 1.413333333 |

| 8-0 | 3.504 | 1.872 | 1.856 | 2.410666667 |  |  |
| --- | --- | --- | --- | --- | --- | --- |
| 8-1 | 1.104 | 1.04 | 1.52 | 1.221333333 |  |  |
| 8-3 | 1.12 | 1.184 | 0.688 | 0.997333333 |  |  |
| 8-5 | 2.432 | 0.608 | 1.968 | 1.669333333 |  |  |
| 8-7 | 1.408 | 1.536 | 1.472 | 1.472 |  |  |
|  |  |  |  |  |  |  |
| 10-0 | 2.32 | 2.576 | 1.552 | 2.149333333 |  |  |
| 10-1 | 3.904 | 4.032 | 3.168 | 3.701333333 |  |  |
| 10-3 | 0.784 | 0.832 | 1.216 | 0.944 |  |  |
| 10-5 | 2.096 | 2.448 | 2.352 | 2.298666667 |  |  |
| 10-7 | 0.608 | 0.64 | 2.992 | 1.413333333 |  |  |
|  |  |  |  |  |  |  |
|  |  |  |  |  |  |  |
|  |  |  | The letter marks indicate the result |  |  |  |
| treat | average | SE | treat | average | 5%significant levels |  |
| 8-0 | 2.4107 | 0.5467 | 10--1 | 3.7013 | a |  |
| 8-1 | 1.2213 | 0.1505 | 8-0 | 2.4107 | b |  |
| 8-3 | 0.9973 | 0.1558 | 10--5 | 2.2987 | b |  |
| 8-5 | 1.6693 | 0.5473 | 10-0 | 2.1493 | bc |  |
| 8-7 | 1.472 | 0.037 | 8--5 | 1.6693 | bc |  |
| 10-0 | 2.1493 | 0.3077 | 8--7 | 1.472 | bc |  |
| 10-1 | 3.7013 | 0.2692 | 10--7 | 1.4133 | bc |  |
| 10-3 | 0.944 | 0.1367 | 8--1 | 1.2213 | bc |  |
| 10-5 | 2.2987 | 0.1051 | 8--3 | 0.9973 | c |  |
| 10-7 | 1.4133 | 0.7894 | 10--3 | 0.944 | c |  |
|  |  |  |  |  |  |  |
|  | 0 | 1 | 3 | 5 | 7 |  |
| Baimi 8 | 2.410666667 | 1.221333333 | 0.997333333 | 1.669333333 | 1.472 |  |
| Baimi 10 | 2.149333333 | 3.701333333 | 0.944 | 2.298666667 | 1.413333333 |  |
|  |  |  |  |  |  |  |
