# Supplementary material for: Evaluation of waterlogging tolerance and responses of protective enzymes to waterlogging stress in pumpkin
Source: PeerJ. 2023 Apr 21;11:e15177. doi: 10.7717/peerj.15177 (PMC10124548; doi:10.7717/peerj.15177)
Supplement: Supplemental Information 9 [file peerj-11-15177-s009.docx]

|  | Pos | Cp1 | average1 | Cp2 | average2 | △0CT1 | △CT2 | △△CT | N（扩增倍数） | average3 |  |
| --- | --- | --- | --- | --- | --- | --- | --- | --- | --- | --- | --- |
|  |  |  |  |  |  | △0CT1= average2 -average1 | △CT2= Cp2-Cp1 | △△CT=△CT2-△0CT1 | N=2-△△CT |  |  |
| sod | 10-0 | 25.65 | 25.45 | 25.81 | 25.80333333 | 0.353333333 |  | 0 | 1 | 1 |  |
|  |  | 25.44 |  | 25.83 |  |  |  |  |  |  |  |
|  |  | 25.26 |  | 25.77 |  |  |  |  |  |  |  |
|  | 10-1 | 25.21 |  | 23.19 |  |  | -2.0200 | -2.373333333 | 5.181369008 | 5.066590651 |  |
|  |  | 25.2 |  | 23.29 |  |  | -1.91 | -2.263333333 | 4.800994667 |  |  |
|  |  | 25.28 |  | 23.25 |  |  | -2.03 | -2.383333333 | 5.217408279 |  |  |
|  | 10-3 | 26.32 |  | 22.84 |  |  | -3.48 | -3.833333333 | 14.25437949 | 18.00846254 |  |
|  |  | 26.74 |  | 22.81 |  |  | -3.93 | -4.283333333 | 19.47205622 |  |  |
|  |  | 26.84 |  | 22.85 |  |  | -3.99 | -4.343333333 | 20.2989519 |  |  |
|  | 10-5 | 26.88 |  | 24.55 |  |  | -2.33 | -2.683333333 | 6.423383055 | 6.297484268 |  |
|  |  | 26.75 |  | 24.54 |  |  | -2.21 | -2.563333333 | 5.910717762 |  |  |
|  |  | 26.92 |  | 24.56 |  |  | -2.36 | -2.713333333 | 6.558351987 |  |  |
|  | 10-7 | 27.97 |  | 26.66 |  |  | -1.31 | -1.663333333 | 3.167475221 | 3.268494763 |  |
|  |  | 28.31 |  | 26.64 |  |  | -1.67 | -2.023333333 | 4.065219729 |  |  |
|  |  | 28.52 |  | 27.51 |  |  | -1.01 | -1.363333333 | 2.572789339 |  |  |
|  | 8-0 | 28.65 | 28.61333333 | 25.89 | 25.86 | -2.753333333 |  | 0 | 1 | 1 |  |
|  |  | 28.63 |  | 25.92 |  |  |  |  |  |  |  |
|  |  | 28.56 |  | 25.77 |  |  |  |  |  |  |  |
|  | 8-1 | 26.57 |  | 23.82 |  |  | -2.75 | 0.003333333 | 0.997692177 | 1.110515654 |  |
|  |  | 26.73 |  | 23.81 |  |  | -2.92 | -0.166666667 | 1.122462048 |  |  |
|  |  | 26.87 |  | 23.84 |  |  | -3.03 | -0.276666667 | 1.211392737 |  |  |
|  | 8-3 | 29.7 |  | 24.92 |  |  | -4.78 | -2.026666667 | 4.07462324 | 3.045473744 |  |
|  |  | 29.74 |  | 24.89 |  |  | -4.85 | -2.096666667 | 4.277199994 |  |  |
|  |  | 29.29 |  | 24.93 |  |  | -4.36 | -1.606666667 | 3.045473744 |  |  |
|  | 8-5 | 28.2 |  | 25.67 |  |  | -2.53 | 0.223333333 | 0.856584019 | 0.825350995 |  |
|  |  | 28.18 |  | 25.58 |  |  | -2.6 | 0.153333333 | 0.899170536 |  |  |
|  |  | 28.14 |  | 25.86 |  |  | -2.28 | 0.473333333 | 0.720298431 |  |  |
|  | 8-7 | 32.21 |  | 29.94 |  |  | -2.27 | 0.483333333 | 0.715322966 | 0.774033136 |  |
|  |  | 32.41 |  | 30.06 |  |  | -2.35 | 0.403333333 | 0.75610928 |  |  |
|  |  | 32.74 |  | 30.22 |  |  | -2.52 | 0.233333333 | 0.850667161 |  |  |

|  | Pos | Cp1 | average1 | Cp2 | average2 | △0CT1 | △CT2 | △△CT | N（扩增倍数） | average3 |
| --- | --- | --- | --- | --- | --- | --- | --- | --- | --- | --- |
|  |  |  |  |  |  | △0CT1= average2 -average1 | △CT2= Cp2-Cp1 | △△CT=△CT2-△0CT1 | N=2-△△CT |  |
| pod | 8-0 | 28.65 | 28.61333333 | 24.69 | 24.66666667 | -3.946666667 |  | 0 | 1 | 1 |
|  |  | 28.63 |  | 24.79 |  |  |  |  |  |  |
|  |  | 28.56 |  | 24.52 |  |  |  |  |  |  |
|  | 8-1 | 26.57 |  | 21.7 |  |  | -4.87 | -0.923333333 | 1.896492062 | 2.086205452 |
|  |  | 26.73 |  | 21.73 |  |  | -5 | -1.053333333 | 2.075319318 |  |
|  |  | 26.87 |  | 21.73 |  |  | -5.14 | -1.193333333 | 2.286804974 |  |
|  | 8-3 | 26.57 |  | 20.92 |  |  | -5.65 | -1.703333333 | 3.25652507 | 3.056504945 |
|  |  | 26.34 |  | 20.87 |  |  | -5.47 | -1.523333333 | 2.874544437 |  |
|  |  | 26.49 |  | 20.94 |  |  | -5.55 | -1.603333333 | 3.038445328 |  |
|  | 8-5 | 28.2 |  | 22.72 |  |  | -5.48 | -1.533333333 | 2.894538475 | 2.771661176 |
|  |  | 28.18 |  | 22.81 |  |  | -5.37 | -1.423333333 | 2.682044796 |  |
|  |  | 28.14 |  | 22.74 |  |  | -5.4 | -1.453333333 | 2.738400258 |  |
|  | 8-7 | 32.21 |  | 27.08 |  |  | -5.13 | -1.183333333 | 2.271008858 | 2.665172809 |
|  |  | 32.41 |  | 27.16 |  |  | -5.25 | -1.303333333 | 2.467984499 |  |
|  |  | 32.74 |  | 27.09 |  |  | -5.65 | -1.703333333 | 3.25652507 |  |
|  | 10-0 | 25.65 | 25.45 | 23.05 | 23.11666667 | -2.333333333 |  | 0 | 1 | 1 |
|  |  | 25.44 |  | 23.15 |  |  |  |  |  |  |
|  |  | 25.26 |  | 23.15 |  |  |  |  |  |  |
|  | 10-1 | 25.21 |  | 22.75 |  |  | -2.46 | -0.126666667 | 1.091768265 | 1.110183807 |
|  |  | 25.2 |  | 22.76 |  |  | -2.44 | -0.106666667 | 1.076737568 |  |
|  |  | 25.28 |  | 22.73 |  |  | -2.55 | -0.216666667 | 1.162045587 |  |
|  | 10-3 | 26.32 |  | 23.08 |  |  | -3.24 | -0.906666667 | 1.874708993 | 2.369569722 |
|  |  | 26.74 |  | 23.08 |  |  | -3.66 | -1.326666667 | 2.508224819 |  |
|  |  | 26.84 |  | 23.06 |  |  | -3.78 | -1.446666667 | 2.725775354 |  |
|  | 10-5 | 26.12 |  | 22.58 |  |  | -3.54 | -1.206666667 | 2.308037504 | 2.012650162 |
|  |  | 25.67 |  | 22.64 |  |  | -3.03 | -0.696666667 | 1.620755722 |  |
|  |  | 25.92 |  | 22.51 |  |  | -3.41 | -1.076666667 | 2.109157259 |  |
|  | 10-7 | 30.27 |  | 28.61 |  |  | -1.66 | 0.673333333 | 0.627056205 | 0.733558386 |
|  |  | 30.29 |  | 28.58 |  |  | -1.71 | 0.623333333 | 0.649169294 |  |
|  |  | 30.74 |  | 28.52 |  |  | -2.22 | 0.113333333 | 0.92444966 |  |

|  | Pos | Cp1 | average1 | Cp2 | average2 | △0CT1 | △CT2 | △△CT | N（扩增倍数） | average3 |
| --- | --- | --- | --- | --- | --- | --- | --- | --- | --- | --- |
|  |  |  |  |  |  | △0CT1= average2 -average1 | △CT2= Cp2-Cp1 | △△CT=△CT2-△0CT1 | N=2^-△△CT^ |  |
| cat | 8-0 | 28.65 | 28.61333333 | 24.67 | 24.69 | -3.96 |  | 0 | 1 | 1 |
|  |  | 28.63 |  | 24.65 |  |  |  |  |  |  |
|  |  | 28.56 |  | 24.75 |  |  |  |  |  |  |
|  | 8-1 | 26.57 |  | 22.72 |  |  | -3.85 | 0.26 | 0.835087919 | 0.989323935 |
|  |  | 26.73 |  | 22.62 |  |  | -4.11 | 0 | 1 |  |
|  |  | 26.87 |  | 22.58 |  |  | -4.29 | -0.18 | 1.132883885 |  |
|  | 8-3 | 29.7 |  | 22.21 |  |  | -7.49 | -3.38 | 10.41073484 | 8.872078769 |
|  |  | 29.74 |  | 22.27 |  |  | -7.47 | -3.36 | 10.26740718 |  |
|  |  | 29.29 |  | 22.61 |  |  | -6.68 | -2.57 | 5.938094283 |  |
|  | 8-5 | 28.2 |  | 23.08 |  |  | -5.12 | -1.01 | 2.0139111 | 2.075995958 |
|  |  | 28.18 |  | 22.97 |  |  | -5.21 | -1.1 | 2.143546925 |  |
|  |  | 28.14 |  | 22.98 |  |  | -5.16 | -1.05 | 2.070529848 |  |
|  | 8-7 | 32.21 |  | 27.08 |  |  | -5.13 | -1.02 | 2.02791896 | 2.379891409 |
|  |  | 32.41 |  | 27.16 |  |  | -5.25 | -1.14 | 2.203810232 |  |
|  |  | 32.74 |  | 27.09 |  |  | -5.65 | -1.54 | 2.907945035 |  |
|  | 10-0 | 25.65 | 25.45 | 24.54 | 24.49666667 | -0.953333333 |  | 0 | 1 | 1 |
|  |  | 25.44 |  | 24.43 |  |  |  |  |  |  |
|  |  | 25.26 |  | 24.52 |  |  |  |  |  |  |
|  | 10-1 | 25.21 |  | 21.73 |  |  | -3.48 | -2.526666667 | 5.762387447 | 5.776858962 |
|  |  | 25.2 |  | 21.68 |  |  | -3.52 | -2.566666667 | 5.924390209 |  |
|  |  | 25.28 |  | 21.83 |  |  | -3.45 | -2.496666667 | 5.643799228 |  |
|  | 10-3 | 26.32 |  | 22.21 |  |  | -4.11 | -3.156666667 | 8.917669093 | 11.56017791 |
|  |  | 26.74 |  | 22.14 |  |  | -4.6 | -3.646666667 | 12.52437466 |  |
|  |  | 26.84 |  | 22.16 |  |  | -4.68 | -3.726666667 | 13.23848996 |  |
|  | 10-5 | 26.88 |  | 23.16 |  |  | -3.72 | -2.766666667 | 6.805337288 | 6.58919875 |
|  |  | 26.75 |  | 23.22 |  |  | -3.53 | -2.576666667 | 5.965597602 |  |
|  |  | 26.92 |  | 23.16 |  |  | -3.76 | -2.806666667 | 6.99666136 |  |
|  | 10-7 | 30.27 |  | 27.63 |  |  | -2.64 | -1.686666667 | 3.21912069 | 3.484590308 |
|  |  | 30.29 |  | 27.73 |  |  | -2.56 | -1.606666667 | 3.045473744 |  |
|  |  | 30.74 |  | 27.72 |  |  | -3.02 | -2.066666667 | 4.189176491 |  |

|  | Pos | Cp1 | average1 | Cp2 | average2 | △0CT1 | △CT2 | △△CT | N（扩增倍数） | average3 |
| --- | --- | --- | --- | --- | --- | --- | --- | --- | --- | --- |
|  |  |  |  |  |  | △0CT1= average2 -average1 | △CT2= Cp2-Cp1 | △△CT=△CT2-△0CT1 | N=2^-△△CT^ |  |
| APX | 8-0 | 28.65 | 28.61333333 | 28.03 | 28.07333333 | -0.54 |  | 0 | 1 | 1 |
|  |  | 28.63 |  | 28.06 |  |  |  |  |  |  |
|  |  | 28.56 |  | 28.13 |  |  |  |  |  |  |
|  | 8-1 | 26.57 |  | 25.55 |  |  | -1.02 | -0.48 | 1.394743666 | 1.521966419 |
|  |  | 26.73 |  | 25.52 |  |  | -1.21 | -0.67 | 1.591072968 |  |
|  |  | 26.87 |  | 25.67 |  |  | -1.2 | -0.66 | 1.580082624 |  |
|  | 8-3 | 26.57 |  | 26.73 |  |  | 0.16 | 0.7 | 0.615572207 | 0.588835488 |
|  |  | 26.34 |  | 26.69 |  |  | 0.35 | 0.89 | 0.539614118 |  |
|  |  | 26.49 |  | 26.66 |  |  | 0.17 | 0.71 | 0.611320139 |  |
|  | 8-5 | 28.2 |  | 26.71 |  |  | -1.49 | -0.95 | 1.931872658 | 1.740179593 |
|  |  | 28.18 |  | 26.84 |  |  | -1.34 | -0.8 | 1.741101127 |  |
|  |  | 28.14 |  | 26.97 |  |  | -1.17 | -0.63 | 1.547564994 |  |
|  | 8-7 | 32.21 |  | 31.22 |  |  | -0.99 | -0.45 | 1.366040257 | 1.540015437 |
|  |  | 32.41 |  | 31.25 |  |  | -1.16 | -0.62 | 1.536875181 |  |
|  |  | 32.74 |  | 31.42 |  |  | -1.32 | -0.78 | 1.717130873 |  |
|  | 10-0 | 25.65 | 25.45 | 26.42 | 26.44333333 | 0.993333333 |  | 0 | 1 |  |
|  |  | 25.44 |  | 26.51 |  |  |  |  |  |  |
|  |  | 25.26 |  | 26.4 |  |  |  |  |  |  |
|  | 10-1 | 25.21 |  | 24.58 |  |  | -0.63 | -1.623333333 | 3.080860445 | 3.306124936 |
|  |  | 25.2 |  | 24.44 |  |  | -0.76 | -1.753333333 | 3.371366179 |  |
|  |  | 25.28 |  | 24.48 |  |  | -0.8 | -1.793333333 | 3.466148183 |  |
|  | 10-3 | 26.32 |  | 24.64 |  |  | -1.68 | -2.673333333 | 6.379013507 | 7.776943412 |
|  |  | 26.74 |  | 24.66 |  |  | -2.08 | -3.073333333 | 8.417158786 |  |
|  |  | 26.84 |  | 24.74 |  |  | -2.1 | -3.093333333 | 8.534657944 |  |
|  | 10-5 | 26.88 |  | 27.02 |  |  | 0.14 | -0.853333333 | 1.806670402 | 1.783272104 |
|  |  | 26.75 |  | 26.99 |  |  | 0.24 | -0.753333333 | 1.68568309 |  |
|  |  | 26.92 |  | 27.02 |  |  | 0.1 | -0.893333333 | 1.85746282 |  |
|  | 10-7 | 30.27 |  | 29.88 |  |  | -0.39 | -1.383333333 | 2.60870414 | 2.980776863 |
|  |  | 30.29 |  | 30 |  |  | -0.29 | -1.283333333 | 2.434007027 |  |
|  |  | 30.74 |  | 29.77 |  |  | -0.97 | -1.963333333 | 3.899619423 |  |

| SOD | 百蜜8号 |  |  | 百蜜10号 |  |  |  |
| --- | --- | --- | --- | --- | --- | --- | --- |
| 0 | 1 | 1 | 1 | 1 | 1 | 1 |  |
| 1 | 0.997692177 | 1.122462048 | 1.211392737 | 5.181369008 | 4.800994667 | 5.217408279 |  |
| 3 | 4.07462324 | 4.277199994 | 3.045473744 | 14.25437949 | 19.47205622 | 20.2989519 |  |
| 5 | 0.856584019 | 0.899170536 | 0.720298431 | 6.423383055 | 5.910717762 | 6.558351987 |  |
| 7 | 0.715322966 | 0.75610928 | 0.850667161 | 3.167475221 | 4.065219729 | 2.572789339 |  |
|  |  |  |  |  |  |  |  |
|  |  |  |  |  |  |  |  |
| The letter marks indicate the result |  |  |  |  |  |  |  |
| treat | average | 5%significant levels | treat | average | SE |  |  |
| 10--3 | 18.0085 | a | 处理1 | 1 | 0 |  |  |
| 10--5 | 6.2975 | b | 处理2 | 1.1105 | 0.1074 |  |  |
| 10--1 | 5.0666 | bc | 处理3 | 3.7991 | 0.6605 |  |  |
| 8--3 | 3.7991 | c | 处理4 | 0.8254 | 0.0935 |  |  |
| 10--7 | 3.2685 | c | 处理5 | 0.774 | 0.0695 |  |  |
| 8--1 | 1.1105 | d | 处理6 | 1 | 0 |  |  |
| 8-0 | 1 | d | 处理7 | 5.0666 | 0.2307 |  |  |
| 10-0 | 1 | d | 处理8 | 18.0085 | 3.2773 |  |  |
| 8--5 | 0.8254 | d | 处理9 | 6.2975 | 0.3417 |  |  |
| 8--7 | 0.774 | d | 处理10 | 3.2685 | 0.7513 |  |  |
|  |  |  |  |  |  |  |  |
|  |  |  |  |  |  |  |  |
|  | 0 | 1 | 3 | 5 | 7 |  |  |
| Baimi 8 | 1 | 1.110515654 | 3.045473744 | 0.825350995 | 0.774033136 |  |  |
| Baimi 10 | 1 | 5.066590651 | 18.00846254 | 6.297484268 | 3.268494763 |  |  |

| POD |  | 百蜜8号 |  |  | 百蜜10号 |  |  |
| --- | --- | --- | --- | --- | --- | --- | --- |
|  | 8-0 | 1 | 1 | 1 | 1 | 1 | 1 |
|  | 8-1 | 1.896492062 | 2.075319318 | 2.286804974 | 1.091768265 | 1.076737568 | 1.162045587 |
|  | 8-3 | 3.25652507 | 2.874544437 | 3.038445328 | 1.874708993 | 2.508224819 | 2.725775354 |
|  | 8-5 | 2.894538475 | 2.682044796 | 2.738400258 | 2.308037504 | 1.620755722 | 2.109157259 |
|  | 8-7 | 2.271008858 | 2.467984499 | 3.25652507 | 0.627056205 | 0.649169294 | 0.92444966 |
|  |  |  |  |  |  |  |  |
|  | The letter marks indicate the result |  |  |  |  |  |  |
|  | treat | average | 5%significant levels | treat | average | SE |  |
|  | 处理3 | 3.0565 | a | 处理1 | 1 | 0 |  |
|  | 处理4 | 2.7716 | ab | 处理2 | 2.0862 | 0.1954 |  |
|  | 处理5 | 2.6652 | ab | 处理3 | 3.0565 | 0.1916 |  |
|  | 处理8 | 2.3696 | bc | 处理4 | 2.7716 | 0.1101 |  |
|  | 处理2 | 2.0862 | c | 处理5 | 2.6652 | 0.5215 |  |
|  | 处理9 | 2.0127 | c | 处理6 | 1 | 0 |  |
|  | 处理7 | 1.1102 | d | 处理7 | 1.1102 | 0.0455 |  |
|  | 处理6 | 1 | d | 处理8 | 2.3696 | 0.4422 |  |
|  | 处理1 | 1 | d | 处理9 | 2.0127 | 0.3536 |  |
|  | 处理10 | 0.7336 | d | 处理10 | 0.7336 | 0.1656 |  |
|  |  |  |  |  |  |  |  |
|  |  |  |  |  |  |  |  |
|  |  | 0 | 1 | 3 | 5 | 7 |  |
|  | Baimi 8 | 1 | 2.086205452 | 3.056504945 | 2.771661176 | 2.665172809 |  |
|  | Baimi 10 | 1 | 1.110183807 | 2.369569722 | 2.012650162 | 0.733558386 |  |

|  | CAT | 百蜜8号 |  |  | 百蜜10号 |  |  |  |  |
| --- | --- | --- | --- | --- | --- | --- | --- | --- | --- |
|  | 0 | 1 | 1 | 1 | 1 | 1 | 1 |  |  |
|  | 1 | 0.835087919 | 1 | 1.132883885 | 5.762387447 | 5.924390209 | 5.643799228 |  |  |
|  | 3 | 10.41073484 | 10.26740718 | 5.938094283 | 8.917669093 | 12.52437466 | 13.23848996 |  |  |
|  | 5 | 2.0139111 | 2.143546925 | 2.070529848 | 6.805337288 | 5.965597602 | 6.99666136 |  |  |
|  | 7 | 2.02791896 | 2.203810232 | 2.907945035 | 3.21912069 | 3.045473744 | 4.189176491 |  |  |
|  |  |  |  |  |  |  |  |  |  |
|  |  | The letter marks indicate the result |  |  |  |  |  |  |  |
|  |  | treat | average | 5%significant levels |  | 处理 | 均值 | 标准差 |  |
|  |  | 处理8 | 11.5602 | a |  | 处理1 | 1 | 0 |  |
|  |  | 处理3 | 8.8721 | b |  | 处理2 | 0.9893 | 0.1492 |  |
|  |  | 处理9 | 6.5892 | c |  | 处理3 | 8.8721 | 2.5419 |  |
|  |  | 处理7 | 5.7769 | c |  | 处理4 | 2.076 | 0.065 |  |
|  |  | 处理10 | 3.4846 | d |  | 处理5 | 2.3799 | 0.4657 |  |
|  |  | 处理5 | 2.3799 | de |  | 处理6 | 1 | 0 |  |
|  |  | 处理4 | 2.076 | de |  | 处理7 | 5.7769 | 0.1409 |  |
|  |  | 处理1 | 1 | e |  | 处理8 | 11.5602 | 2.3162 |  |
|  |  | 处理6 | 1 | e |  | 处理9 | 6.5892 | 0.5485 |  |
|  |  | 处理2 | 0.9893 | e |  | 处理10 | 3.4846 | 0.6163 |  |
|  |  |  |  |  |  |  |  |  |  |
|  |  |  |  |  |  |  |  |  |  |
|  |  | 0 | 1 | 3 | 5 | 7 |  |  |  |
|  | Baimi 8 | 1 | 0.989323935 | 8.872078769 | 2.075995958 | 2.379891409 |  |  |  |
|  | Baimi 10 | 1 | 5.776858962 | 11.56017791 | 6.58919875 | 3.484590308 |  |  |  |
|  |  |  |  |  |  |  |  |  |  |

|  | APX | 百蜜8号 |  |  | 百蜜10号 |  |  |  |
| --- | --- | --- | --- | --- | --- | --- | --- | --- |
|  | 0 | 1 | 1 | 1 | 1 |  | 1 | 1 |
|  | 1 | 1.394743666 | 1.591072968 | 1.580082624 | 3.080860445 |  | 3.371366179 | 3.466148183 |
|  | 3 | 0.615572207 | 0.539614118 | 0.611320139 | 6.379013507 |  | 8.417158786 | 8.534657944 |
|  | 5 | 1.931872658 | 1.741101127 | 1.547564994 | 1.806670402 |  | 1.68568309 | 1.85746282 |
|  | 7 | 1.366040257 | 1.536875181 | 1.717130873 | 2.60870414 |  | 2.434007027 | 3.899619423 |
|  |  |  |  |  |  |  |  |  |
|  |  |  |  |  |  |  |  |  |
|  |  |  |  |  |  |  |  |  |
|  |  |  |  |  |  |  |  |  |
|  | The letter marks indicate the result |  |  |  |  |  |  |  |
|  | treat | average | 5%significant levels | treat |  |  | average | SE |
|  | 处理8 | 7.777 | a | 处理1 |  |  | 1 | 0 |
|  | 处理7 | 3.3061 | b | 处理2 |  |  | 1.522 | 0.1104 |
|  | 处理10 | 2.9808 | b | 处理3 |  |  | 0.5888 | 0.0427 |
|  | 处理9 | 1.7833 | c | 处理4 |  |  | 1.7402 | 0.1922 |
|  | 处理4 | 1.7402 | c | 处理5 |  |  | 1.54 | 0.1756 |
|  | 处理5 | 1.54 | c | 处理6 |  |  | 1 | 0 |
|  | 处理2 | 1.522 | c | 处理7 |  |  | 3.3061 | 0.2007 |
|  | 处理1 | 1 | cd | 处理8 |  |  | 7.777 | 1.2121 |
|  | 处理6 | 1 | cd | 处理9 |  |  | 1.7833 | 0.0883 |
|  | 处理3 | 0.5888 | d | 处理10 |  |  | 2.9808 | 0.8005 |
|  |  |  |  |  |  |  |  |  |
|  |  | 0 | 1 | 3 | 5 |  | 7 |  |
|  | Baimi 8 | 1 | 1.521966419 | 0.588835488 | 1.740179593 |  | 1.540015437 |  |
|  | Baimi 10 | 1 | 3.306124936 | 7.776943412 | 1.783272104 |  | 2.980776863 |  |
